# G-CSF Predicts Cardiovascular Events in Patients with Stable Coronary Artery Disease

**DOI:** 10.1371/journal.pone.0142532

**Published:** 2015-11-10

**Authors:** Katharina M. Katsaros, Walter S Speidl, Svitlana Demyanets, Stefan P. Kastl, Konstantin A. Krychtiuk, Anna Wonnerth, Gerlinde Zorn, Ioannis Tentzeris, Serdar Farhan, Gerald Maurer, Johann Wojta, Kurt Huber

**Affiliations:** 1 Department of Internal Medicine II, Cardiology, Medical University of Vienna, Vienna, Austria; 2 Ludwig Boltzmann Cluster for Cardiovascular Research, Vienna, Austria; 3 3rd Medical Department, Cardiology and Intensive Care Medicine, Wilhelminenhospital, Vienna, Austria; University of Colorado Denver, UNITED STATES

## Abstract

Granulocyte-colony-stimulating-factor (G-CSF) induces mobilization of progenitor cells but may also exert pro-inflammatory and pro-thrombotic effects. Treatment with recombinant G-CSF after acute myocardial infarction is currently under examination and has been associated with in-stent restenosis. However, it is not known whether plasma levels of endogenous G-CSF are also associated with an increased cardiovascular risk. Therefore we included 280 patients with angiographically proven stable coronary artery disease. G-CSF was measured by specific ELISA and patients were followed for a median of 30 months for the occurrence of major adverse cardiovascular events (MACE: death, myocardial infarction, re-hospitalization). Those with cardiac events during follow-up showed significant higher G-CSF levels (32.3 pg/mL IQR 21.4–40.5 pg/mL vs. 24.6 pg/mL IQR 16.4–34.9 pg/mL; p<0.05) at baseline. Patients with G-CSF plasma levels above the median had a 2-fold increased risk for MACE (p<0.05). This was independent from established cardiovascular risk factors. In addition, G-CSF above the median was a predictor of clinical in-stent restenosis after implantation of bare-metal stents (6.6% vs. 19.4%; p<0.05) but not of drug-eluting stents (7.7% vs. 7.6%; p = 0.98). This data suggests that endogenous plasma levels of G-CSF predict cardiovascular events independently from established cardiac risk factors and are associated with increased in-stent restenosis rates after implantation of bare metal stents.

## Background

Granulocyte-colony-stimulating-factor (G-CSF) is produced by monocytes, macrophages, fibroblasts, endothelial cells and bone marrow stromal cells, respectively. It induces mobilization of progenitor cells and acts as proliferative agent for stem cells. On the other side G-CSF has well known pro-inflammatory properties, which might be explained due to its ability in controlling neutrophil numbers as well as their activity during inflammation.[1] As a clinical consequence of inflammatory mechanisms instent-restenosis but also progression of underlying coronary artery disease (CAD) might occur.[2] Further—as platelets are known to express G-CSF receptors—rising G-CSF plasma levels might result in a hyper-coagulable state due to increased platelet aggregation[3], which in turn might contribute to stent thrombosis, myocardial infarction and sudden death.[2]

Treatment with recombinant G-CSF has been under examination after myocardial infarction and a large number of clinical trials have tested its effects on myocardial infarct size.[4] It is suspected to prevent left ventricular remodeling and dysfunction after myocardial infarction; it may improve chronic myocardial ischemia and stimulates re-endothelialization after vessel-injury in animal models. In contrast to these beneficial aspects, there have also been safety concerns regarding an increase of in-stent restenosis in patients receiving recombinant G-CSF.[4]

However, despite various studies using recombinant G-CSF, it is not known whether plasma levels of circulating endogenous G-CSF are associated with an increased cardiovascular (CV) risk. Therefore, it was our aim to evaluate whether endogenous G-CSF plasma levels are associated with CV events and the occurrence of in-stent restenosis in patients with stable CAD.

## Methods

### Patients

We included consecutive 280 patients with angiographically proven stable CAD undergoing elective stent implantation for stable angina pectoris. Patients with unstable angina or myocardial infarction within the previous six months were not included. PCI and stent implantation (PCIs) was performed according to standard techniques and stent type was left to the decision of the experienced interventionalist. Study subjects received a loading dose of 600 mg clopidogrel pre-PCIs. Intravenous unfractionated heparin was administered as to standard practice. After the procedure, patients were maintained on aspirin 100 mg indefinitely, and clopidogrel 75 mg according to the guidelines of the European Society of Cardiology (ESC) (ref). Other medications such as beta-blockers and angiotensin-converting-enzyme inhibitors were given as appropriate. Statin therapy was routinely administered to all patients. The study was approved by the institutional ethics committee for human subjects (EK 10-046-VK_NZ).

### Sample collection

Blood from the cubital vein was taken under fasting conditions immediately before PCIs into serum seperator tubes. Samples were allowed to clot for 30 minutes (min), were then centrifugated at 1500g for 15min and stored at −80°C until use. G-CSF was measured by specific ELISA (R&D Systems, Minneapolis, MN), according to the manufacturer’s instructions. Laboratory determinations were performed by investigators that were blinded to clinical characteristics and patients' outcome.

### Follow-up

Patients were followed for a median of 30 (20–38) months for the occurrence of a combined clinical endpoint (MACE, including all cause death, myocardial infarction [5] and re-hospitalization for cardiac causes). No patients were lost to follow-up.

### Statistical analysis

We estimated a MACE rate of 15% over a median follow-up of 30 months in this cohort of CAD patients. Therefore, calculation of sample size revealed that we would need at least 245 patients to detect a difference in G-CSF serum levels of 20% with a power of 80% and significance level (two-tailed) of 0.05.[6] Continuous variables are expressed as mean ± SD or as median (interquartile range). Categorical variables are summarized as counts and percentages and were compared by the chi-square or by Fisher exact test as appropriate. Continuous variables were compared using Student's t-test when normally distributed and by Mann-Whitney-U test when not normally distributed. Spearman correlation was used to determine the correlation between level of G-CSF and cardiovascular risk factors. A multivariate Cox proportional hazards model was applied to assess the effect of G-CSF on event-free survival, giving hazard ratios (HR) and 95% confidence intervals (95% CI). The combined endpoint was used as dependent variable and potentially confounding baseline variables were used as independent variables. Baseline variables were selected for the model if they a) had either a clinically plausible relation with the outcome or b) appeared to be imbalanced between patients with and without clinical event indicated by a p-value <0.20. Event-free survival rates until the first combined event or until death are presented as Kaplan Meier curves and compared by means of the Log Rank test. A value of p < 0.05 (two-tailed) was considered statistically significant. All statistical analyses were performed with the statistical software package SPSS version 18.0 (SPSS, Inc., Chicago, Illinois).

## Results

Baseline characteristics of study population are given in Table 1. The mean age was 64.5±11.1 years and 74.3% of patients were male. MACE occurred in 46 (16.4%) patients and 19 (6.8%) patients died during follow-up. G-CSF showed a small trend to increase with age (R = 0.11; p = 0.057) but was not associated with other baseline characteristics or CV risk factors. Angiographical and interventional characteristics of patients with and without combined endpoint during follow-up period are given in Table 2.

**Table 1:** Baseline characteristics of patients with and without combined endpoint during follow up period.

	Total	MACE	No MACE	p-Value
n	280	46	243	
Age (years)	64.5±11.1	68.1±11.8	63,8±10.9	0.02
Sex (male)	208 (74.3%)	33 (71.8%)	175 (74.8%)	0.71
Hypertension, N (%)	219 (78.2%)	34 (73.9%)	185 (79.1%)	0.44
Diabetes, N (%)	65 (23.2%)	12 (26.1%)	53 (22.6%)	0.70
Family history, N (%)	22 (7.9%)	2 (4.3%)	20 (8.5%	0.55
Smoker, N (%)	68 (24.3%)	10(21.7%)	58 (24.8%)	0.47
BMI (kg/m^2^)	28.0±4.6	28.1±4.9	27.9±4.5	0.85
Hyperlipidemia, N (%)	212 (75.7%)	30 (65.2%)	182 (77.8%)	0.09
Previous MCI, N (%)	78 (27.9%)	21 (45.7%)	57 (24.4%)	0.003

Values are given as mean±SD or n (%); MACE: major adverse cardiovascular events (death, myocardial infarction, rehospitalisation); BMI: body mass index; MCI myocardial infarction.

**Table 2:** Angiographical and interventional characteristics of patients with and without combined endpoint during follow-up period.

	Total	MACE	No MACE	p-value
n	280	46	243	
CAD extent (1VD/2VD/3VD)	(139/80/61)	(19/13/14)	(120/66/42)	0.26
Target Vessel				
LM	2 (0.7%)	1 (2%)	1 (0.4%)	0.11
LAD	132 (47%)	17 (37%)	115 (49%)	
CX	67 (24%)	9 (19%)	58 (25%)	
RCA	77 (28%)	19 (42%)	58 (25%)	
Number of stents	1.37±0.65	1.46±0.48	1.35±4.0.62	0.33
Type of stent				
BMS	123 (44%)	26 (56%)	97 (42%)	0.06
DES	157 (56%)	20 (44%)	137 (58%)	

MACE: major adverse cardiac cardiovascular events (death, myocardial infarction, rehospitalisation); CAD: coronary artery disease; BMS: bare metal stent; DES: drug eluting stent.

**Fig 1:**
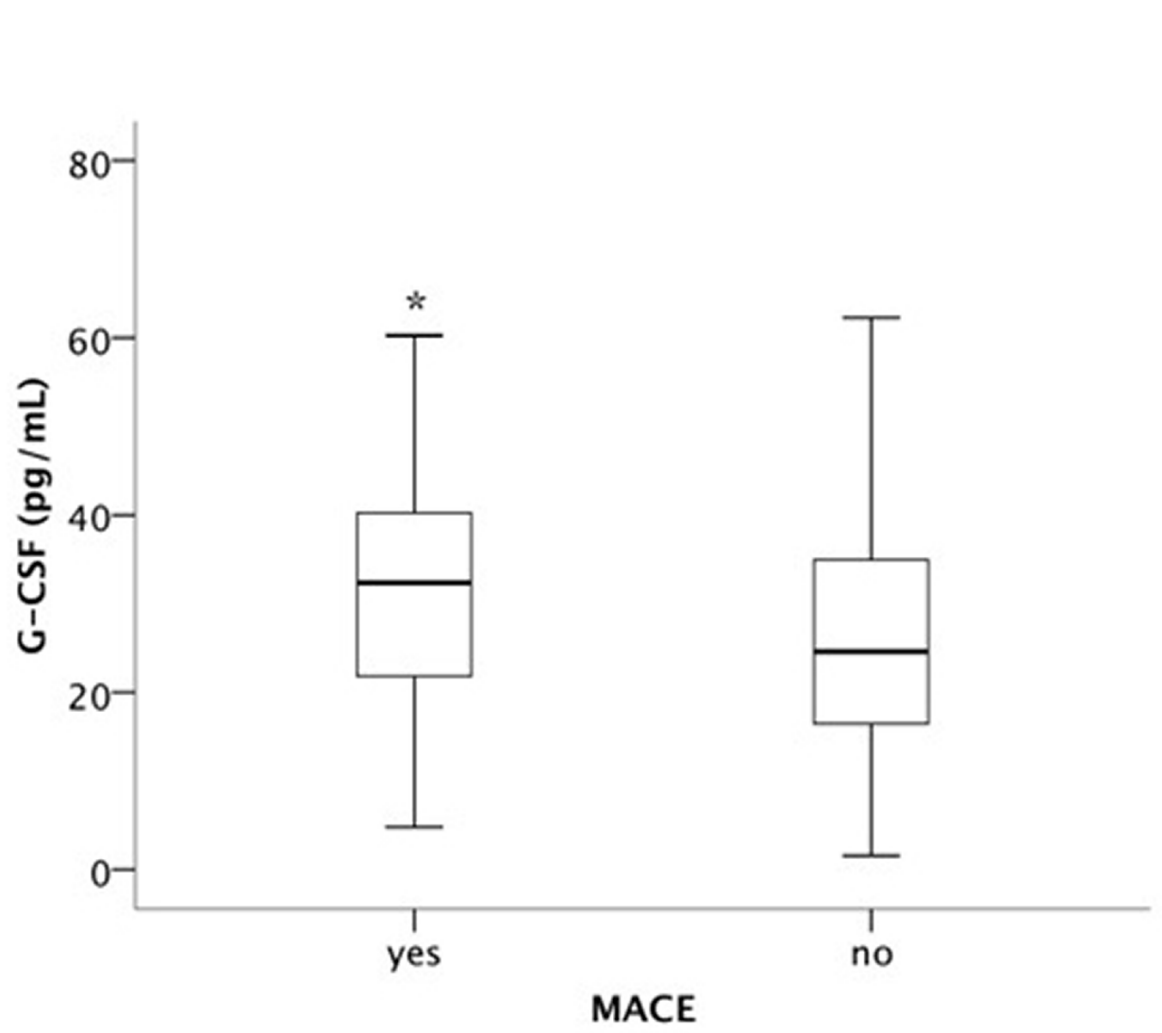
Median G-CSF levels for patients with or without combined endpoint (all-cause death, myocardial infarction or re-hospitalization) during follow up. * p<0.05.

**Fig 2:**
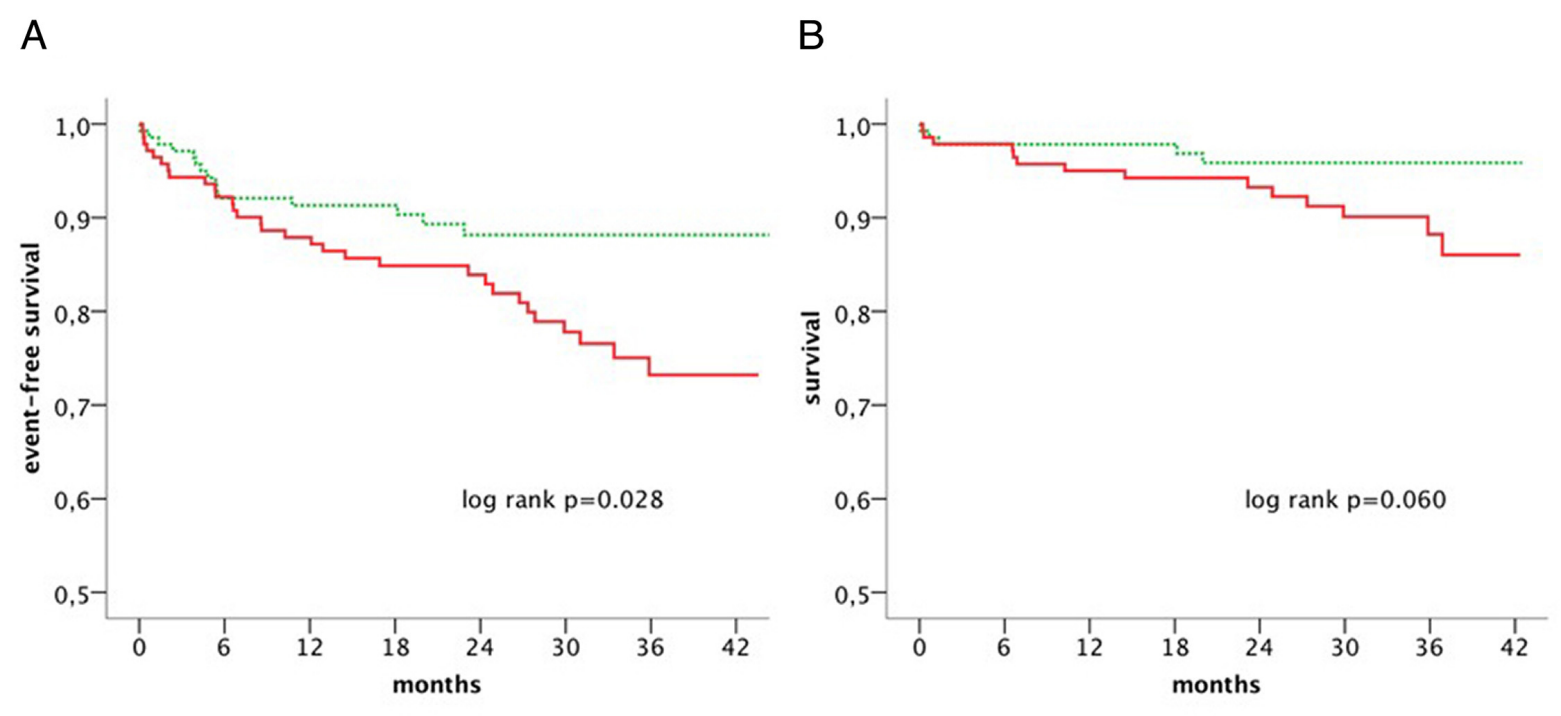
Cumulative CV event-free survival (all-cause death, myocardial infarction and rehospitalization; A) and cumulative survival (all-cause mortality; B) for patients with G-CSF below the median (green, dotted line) and above the median (red, full line).

Patients with CV events during follow-up showed significantly higher endogenous G-CSF levels before PCIs (32.3 pg/mL IQR 21.4–40.5 pg/mL vs. 24.6 pg/mL IQR 16.4–34.9 pg/mL; p<0.05). Median G-CSF levels for patients with or without MACE during follow up are shown in Fig 1. Patients with G-CSF plasma levels above the median had a two-fold increased risk for a CV event (p<0.05). Cox regression analysis revealed that this was independent from established CV risk factors (Table 3). Survival curves for patients with G-CSF plasma levels below the median and above the median for the combined endpoint are shown in Fig 2A, and for all-cause mortality in Fig 2B. All-cause mortality showed a trend to be increased in patients with G-CSF above the median as compared to G-CSF plasma levels below the median (p = 0.06), respectively.

**Table 3:** Multivariate Cox proportional hazards model assessing the association between G-CSF (below and above median) and major adverse cardiovascular events (death, myocardial infarction and rehospitalisation).

	Laboratory Range	Hazard Ratio	95% confidence interval	p-value
**Univariate Model**				
G-CSF	<25.6 pg/mL	1.0	-	-
G-CSF	25.6 to 145.3 pg/mL	1.97	1.06 to 3.65	0.031
**Model adjusts for age, gender, hyperlipidemia, history of myocardial infarction**				
G-CSF	<25.6 pg/mL	1.0	-	-
G-CSF	25.6 to 145.3 pg/mL	1.88	1.01 to 3.49	0.046

**-** denotes reference category.

In addition, G-CSF plasma levels above the median were associated with a higher rate of in-stent restenosis in patients with bare-metal stents (BMS; 6.6% vs. 19.4%; p<0.05; n = 123) but not in patients with drug-eluting stents (DES; 7.7% vs. 7.6%; p = 0.98; n = 157).

## Discussion

We were able to show for the first time that pre-existing plasma levels of G-CSF predict MACE in patients with stable CAD, independently from established CV risk factors. Furthermore, endogenous G-CSF levels predict clinical in-stent restenosis after implantation of BMS, but not after implantation of DES.

While other colony-stimulating factors like M-CSF and GM-CSF have been extensively studied in animal and clinical studies of atherosclerotic diseases, G-CSF has received hardly any attention and results are conflicting.[7] Addition of G-CSF to atherosclerosis-prone rabbits[8] and ApoE-/-mice[9] reduced atherogenesis, whereas conversely increased atherosclerotic lesion size in a study of Haghihat et all in ApeE-/-, possibly by increasing *vasa vasorum* neovascularization.[10] In addition, G-CSF acts pro-inflammatory by increasing neutrophil numbers and activity.[1] In the last years, a proatherogenic role of neutrophils have gained attention.[11] Recently, it has been shown that neutrophils within carotid plaques interleuk-8, vascular endothelial growth factor and elastase, which are crucial for plaque development and progression.[12] In addition, increased number of circulating neutrophils have been shown to predict risk of future cardiovascular events in patients with stable and unstable coronary syndroms. [13] In our study, G-CSF plasma levels above the median were associated with a 2-fold increased risk for the occurrence of all-cause death, myocardial infarction or rehospitalization during the follow-up period. In addition, all-cause mortality showed a trend to be increased in patients with G-CSF above the median as compared to G-CSF plasma levels below the median.

Animal studies have shown that G-CSF might prevent restenosis by acceleration of re-endothelialization and a decrease of neo-intima formation after vascular injury [8,14]; However, it has also been shown that G-CSF induces migration of vascular smooth muscle cells.[15] We were able to demonstrate that endogenous G-CSF levels above the median are associated with an increased rate of clinical restenosis in BMS. Our results relating high enodgenous G-CSF levels and restenosis are in line with clinical studies which investigated G-CSF-treatment after myocardial infarction that demonstrated a higher rate of in-stent restenosis or had to be stopped due to a unexpectedly high rate of in-stent restenosis after G-CSF treatment.[16–19] Interestingly, in our study endogenous G-CSF levels were not associated with in-stent restenosis in patients undergoing DES implantation that may locally inhibit smooth muscle cell migration and proliferation.[20–23]

### Limitations and Strengths

Some limitations of the present study have to be acknowledged. First, our study is of observational nature. Accordingly, our results may be explained by unmeasured confounding factors. Therefore, we tried to control for baseline imbalances by multivariate modeling. However, the possibility of residual or undetected confounding is small but cannot be ruled out completely. Although inflammation is a well-known contributor to atherosclerosis and G-CSF is known to increase numbers of neutrophils, differential blood count was not available in our patients, therefore it is not possible to analyse whether the observed effects could have been mediated through circulating neutrophils. On the other hand this investigation represents high-quality as patients were included consecutively and loss-to-follow-up was less than 1%.

### Conclusion

In conclusion, endogenous plasma levels of G-CSF predict CV events independently from established CV risk factors and are associated with in-stent restenosis in patients who underwent BMS implantation.

## References

[1] Hamilton JA (2008) Colony-stimulating factors in inflammation and autoimmunity. Nat Rev Immunol 8: 533–544. 10.1038/nri2356 18551128

[2] Krychtiuk KA, Kastl SP, Speidl WS, Wojta J (2013) Inflammation and coagulation in atherosclerosis. Hamostaseologie 33: 269–282. 10.5482/HAMO-13-07-0039 24043155

[3] Falanga A, Marchetti M, Evangelista V, Manarini S, Oldani E, Giovanelli S, et al (1999) Neutrophil activation and hemostatic changes in healthy donors receiving granulocyte colony-stimulating factor. Blood 93: 2506–2514. 10194429

[4] Zohlnhofer D, Dibra A, Koppara T, de Waha A, Ripa RS, Kastrup J, et al (2008) Stem cell mobilization by granulocyte colony-stimulating factor for myocardial recovery after acute myocardial infarction: a meta-analysis. J Am Coll Cardiol 51: 1429–1437. 10.1016/j.jacc.2007.11.073 18402895

[5] Thygesen K, Alpert JS, Jaffe AS, Simoons ML, Chaitman BR, White HD (2012) Third universal definition of myocardial infarction. Eur Heart J 33: 2551–2567. 10.1093/eurheartj/ehs184 22922414

[6] Dupont WD, Plummer WD Jr (1990) Power and sample size calculations. A review and computer program. Control Clin Trials 11: 116–128. 2161310 10.1016/0197-2456(90)90005-m

[7] Di Gregoli K, Johnson JL (2012) Role of colony-stimulating factors in atherosclerosis. Curr Opin Lipidol 23: 412–421. 10.1097/MOL.0b013e328357ca6e 22964991

[8] Hasegawa H, Takano H, Ohtsuka M, Ueda K, Niitsuma Y, Qin Y, et al (2006) G-CSF prevents the progression of atherosclerosis and neointimal formation in rabbits. Biochem Biophys Res Commun 344: 370–376. 16600176 10.1016/j.bbrc.2006.03.081

[9] Uchiyama R, Hasegawa H, Kameda Y, Ueda K, Kobayashi Y, Komuro I, et al (2012) Role of regulatory T cells in atheroprotective effects of granulocyte colony-stimulating factor. J Mol Cell Cardiol 52: 1038–1047. 10.1016/j.yjmcc.2011.12.016 22285481

[10] Haghighat A, Weiss D, Whalin MK, Cowan DP, Taylor WR (2007) Granulocyte colony-stimulating factor and granulocyte macrophage colony-stimulating factor exacerbate atherosclerosis in apolipoprotein E-deficient mice. Circulation 115: 2049–2054. 17404156 10.1161/CIRCULATIONAHA.106.665570

[11] Baetta R, Corsini A (2010) Role of polymorphonuclear neutrophils in atherosclerosis: current state and future perspectives. Atherosclerosis 210: 1–13. 10.1016/j.atherosclerosis.2009.10.028 19931081

[12] Marino F, Tozzi M, Schembri L, Ferraro S, Tarallo A, Scanzano A, et al (2015) Production of IL-8, VEGF and Elastase by Circulating and Intraplaque Neutrophils in Patients with Carotid Atherosclerosis. PLoS One 10: e0124565 10.1371/journal.pone.0124565 25893670 PMC4404350

[13] Guasti L, Dentali F, Castiglioni L, Maroni L, Marino F, Squizzato A, et al (2011) Neutrophils and clinical outcomes in patients with acute coronary syndromes and/or cardiac revascularisation. A systematic review on more than 34,000 subjects. Thromb Haemost 106: 591–599. 10.1160/TH11-02-0096 21866299

[14] Yoshioka T, Takahashi M, Shiba Y, Suzuki C, Morimoto H, Izawa A, et al (2006) Granulocyte colony-stimulating factor (G-CSF) accelerates reendothelialization and reduces neointimal formation after vascular injury in mice. Cardiovasc Res 70: 61–69. 16448633 10.1016/j.cardiores.2005.12.013

[15] Chen X, Kelemen SE, Autieri MV (2004) AIF-1 expression modulates proliferation of human vascular smooth muscle cells by autocrine expression of G-CSF. Arterioscler Thromb Vasc Biol 24: 1217–1222. 15117732 10.1161/01.ATV.0000130024.50058.de

[16] Mansour S, Vanderheyden M, De Bruyne B, Vandekerckhove B, Delrue L, Van Haute I, et al (2006) Intracoronary delivery of hematopoietic bone marrow stem cells and luminal loss of the infarct-related artery in patients with recent myocardial infarction. J Am Coll Cardiol 47: 1727–1730. 16631016 10.1016/j.jacc.2006.01.039

[17] Kang HJ, Kim HS, Zhang SY, Park KW, Cho HJ, Koo BK, et al (2004) Effects of intracoronary infusion of peripheral blood stem-cells mobilised with granulocyte-colony stimulating factor on left ventricular systolic function and restenosis after coronary stenting in myocardial infarction: the MAGIC cell randomised clinical trial. Lancet 363: 751–756. 15016484 10.1016/S0140-6736(04)15689-4

[18] Morice MC, Serruys PW, Sousa JE, Fajadet J, Ban Hayashi E, Perin M, et al (2002) A randomized comparison of a sirolimus-eluting stent with a standard stent for coronary revascularization. N Engl J Med 346: 1773–1780. 12050336 10.1056/NEJMoa012843

[19] Steinwender C, Hofmann R, Kammler J, Kypta A, Pichler R, Maschek W, et al (2006) Effects of peripheral blood stem cell mobilization with granulocyte-colony stimulating factor and their transcoronary transplantation after primary stent implantation for acute myocardial infarction. Am Heart J 151: 1296 e1297-1213. 16781240 10.1016/j.ahj.2006.03.012

[20] Clever YP, Cremers B, Krauss B, Bohm M, Speck U, Laufs U, et al (2011) Paclitaxel and sirolimus differentially affect growth and motility of endothelial progenitor cells and coronary artery smooth muscle cells. EuroIntervention 7 Suppl K: K32–42. 10.4244/EIJV7SKA6 22027725

[21] Deshpande D, Devalapally H, Amiji M (2008) Enhancement in anti-proliferative effects of paclitaxel in aortic smooth muscle cells upon co-administration with ceramide using biodegradable polymeric nanoparticles. Pharm Res 25: 1936–1947. 10.1007/s11095-008-9614-3 18480968

[22] Blagosklonny MV, Demidenko ZN, Giovino M, Szynal C, Donskoy E, Herrmann RA, et al (2006) Cytostatic activity of paclitaxel in coronary artery smooth muscle cells is mediated through transient mitotic arrest followed by permanent post-mitotic arrest: comparison with cancer cells. Cell Cycle 5: 1574–1579. 16861892 10.4161/cc.5.14.3113

[23] Blagosklonny MV, Darzynkiewicz Z, Halicka HD, Pozarowski P, Demidenko ZN, Barry JJ, et al (2004) Paclitaxel induces primary and postmitotic G1 arrest in human arterial smooth muscle cells. Cell Cycle 3: 1050–1056. 15254417

